# Does manual therapy affect functional and biomechanical outcomes of a sit-to-stand task in a population with low back pain? A preliminary analysis

**DOI:** 10.1186/s12998-019-0290-7

**Published:** 2020-01-24

**Authors:** Giancarlo Carpino, Steven Tran, Stuart Currie, Brian Enebo, Bradley S. Davidson, Samuel J. Howarth

**Affiliations:** 1 Division of Research and Innovation, Canadian Memorial Chiropractic College, Toronto, ON M2H 3 J1 Canada; 2 Department of Mechanical Engineering, University of Denver, Denver, CO USA

**Keywords:** Spinal manipulation, Mobilisation, Activities of daily living, Lumbar spine, Kinematics, Neuromuscular control

## Abstract

**Introduction:**

Manual therapy (MT) hypothetically affects discrepant neuromuscular control and movement observed in populations with low back pain (LBP). Previous studies have demonstrated the limited influence of MT on movement, predominately during range of motion (ROM) testing. It remains unclear if MT affects neuromuscular control in mobility-based activities of daily living (ADLs). The sit-to-stand (STS) task represents a commonly-performed ADL that is used in a variety of clinical settings to assess functional and biomechanical performance.

**Objective:**

To determine whether MT affects functional performance and biomechanical performance during a STS task in a population with LBP.

**Methods:**

Kinematic data were recorded from the pelvis and thorax of participants with LBP, using an optoelectronic motion capture system as they performed a STS task before and after MT from November 2011 to August 2014. MT for each participant consisted of two high-velocity low-amplitude spinal manipulations, as well as two grade IV mobilizations of the lumbar spine and pelvis targeted toward the third lumbar vertebra and sacroiliac joint in a side-lying position; the order of these treatments was randomized. Pelvis and thorax kinematic data were used to derive the time-varying lumbar angle in the sagittal plane for each STS trial. The difference between the maximum and minimum lumbar angles during the STS trial determined the sagittal ROM that was used as the biomechanical outcome. Time to complete each STS trial was used as a functional measure of performance. Pre-MT and post-MT values for the lumbar sagittal ROM and time to completion were statistically analysed using paired samples t-tests.

**Results:**

Data were obtained from 40 participants with 35 useful datasets (NRS = 3.3 ± 1.2; 32.4 ± 9.8 years; 16 females, 19 males). After MT, lumbar sagittal ROM increased by 2.7 ± 5.5 degrees (*p* = 0.007). Time to complete the STS test decreased by 0.4 ± 0.4 s (*p* < 0.001).

**Discussion:**

These findings provide preliminary evidence that MT might influence the biomechanical and functional performance of an STS task in populations with LBP. The MT intervention in this study involved a combination of spinal manipulations and mobilizations. Future work will expand upon these data as a basis for targeted investigations on the effects of either spinal manipulation and mobilization on neuromuscular control and movement in populations with LBP.

## Background

Manual therapies (MTs), including joint mobilisations and spinal manipulation, are effective treatments for some individuals with low back pain (LBP) [1]. Joint mobilisation and manipulation both involve the manual application of force; however, mobilisations are characterised by lower magnitude forces that do not move the joint beyond its physiological range of motion (ROM) whereas manipulations are defined by larger magnitude forces, applied rapidly, that attempt to move the joint beyond its physiological ROM [2]. One hypothesized mechanism of action for MT is related to its potential to impact the neuromechanical function of the spine [3, 4]. Thus, MT could conceivably influence the discrepant motor control strategies and movement patterns observed between those with and without LBP [5, 6]. Previous research on the effects of MT on spine movement has mainly focused on measuring post-treatment changes in movements, such as planar range of motion (ROM), with limited functional relevance [7]. It has yet to be determined if MT applied to the lower back influences movement patterns during a functional task such as those performed during daily living that require coordinated multi-planar and multi-joint movement strategies.

Previous research demonstrates changes in the spine’s mechanical and neuromuscular behaviours following administration of spinal manipulation and joint mobilisation. For example, spinal manipulation decreased paraspinal muscle activation during both quiet lying and full forward spine flexion [8–10], and increased the activity of the internal oblique muscle during rapid arm movements [11]. Neurophysiological work has demonstrated that spinal manipulation influences sensorimotor integration within the central nervous system [12] and can increase both motor unit excitability and cortical drive [13, 14]. Improvement in disability following spinal manipulation has also been associated with a post-treatment decrease in the spine’s posteroanterior stiffness among patients with LBP [15, 16] and an increased thickness of the activated multifidus muscle during an arm raising task with the patient in a prone-lying position [17].

Despite the mechanical and neuromuscular changes, findings on the effect of spinal manipulation and mobilisation on active spine movement have been inconsistent. Millan and colleagues [7] reported in a recent systematic review that spinal manipulation or mobilisation does not change sagittal plane ROM in the lumbar spine. Lehman and McGill [18] also reported no consistent immediate effect of spinal manipulation on ROM for the lumbar spine in any of the three cardinal movement planes in a population of patients with non-specific LBP. Conversely, a secondary analysis of data from a randomized control study of patients with chronic LBP demonstrated changes in spine motion, during a circumduction task, following a 12-week course of spinal manipulative therapy [19]. Recent evidence has reported that cervical ROM increased in neck pain patients following mobilisation applied to the cervical spine [20]. Movements performed by participants in these studies represented non-functional contexts. Regarding the lumbar spine and hip, ROM has demonstrated only weak to moderate correlation with the amount of lumbar and hip movement required to perform certain functional tasks included in activities of daily living, such as the sit-to-stand (STS) task [21]. Studying the biomechanics of functional tasks may provide insight to physical demands that are more challenging and provocative for patients with low back pain, and are more closely associated with the demands from activities of daily living [22].

The STS task, defined by Schenkman and colleagues [23], is a particularly relevant movement for determining functional impairment in patients with LBP. It is frequently performed in daily activities, on average 60 times per day, and requires approximately 60% of a person’s total sagittal plane ROM for the lumbar spine [24, 25]. Previous work has also reported that the STS task, performed repetitively as a clinical test, is a simple and effective tool to objectively evaluate functional impairment [26, 27] and has good test-retest reliability in patients with LBP [28, 29]. Functionally, populations with LBP commonly complain of pain during the STS task [30, 31] and require a longer duration to complete the STS test than healthy populations [32]. Biomechanically, those with LBP have reduced ROM in the lumbar spine and hip joints, with less proportional movement by the lumbar spine [33]. Interjoint coordination between the lumbar spine and hips during the STS task also varies between patients with LBP and healthy participants. Participants with LBP demonstrate less lagging of the hips in the early stage of the STS task and more leading with the hips during the rising phase than control participants [33]. Recent evidence has also demonstrated that patients with LBP perform the STS task in the sagittal plane with a more out of phase movement in the hips and lumbar spine along with considerably more variability from one repetition to the next [34]. These combined functional and biomechanical differences between populations demonstrate the STS task’s utility as a functional evaluation of motor performance in patients with LBP.

Thus, the current study focused on determining if the biomechanical (low back kinematics) and functional (completion time) performance of the STS task changed after a set of MT interventions applied to the lumbar spine and pelvis of participants with acute and chronic LBP. We hypothesized that lumbar sagittal plane ROM during the STS task would increase and that the time to complete the task would decrease after the MT intervention.

## Methods

### Study design

The current study used a pre-experimental single group pretest-posttest design. Raw data were collected at the University of Denver between November 2011 to August 2014 and processed and analysed at the Canadian Memorial Chiropractic College. All protocols for instrumentation and data collection for this investigation were approved by the Colorado Multiple Institutional Review Board (COMIRB #10–1383). These data were obtained as part of a larger study focused on quantifying muscle activities during spinal manipulation in participants with and without LBP [35]. Data processing and analysis procedures were approved by the Canadian Memorial Chiropractic College’s Research Ethics Board (REB #182005).

### Participants

Individuals between the ages of 18–55 with a history of chronic or acute LBP, defined as pain between the lowest rib and the pelvis, were recruited as participants for this investigation. All participants verbally rated their current LBP on an 11-point numerical rating scale (from 0 to 10) on the day of data collection. Inclusion criteria for participants with acute LBP were episodes of LBP lasting less than 3 months duration within the last 4 years and a numerical pain score of at least 2/10 at the time of testing. The chronic pain group was defined as having one or more episodes of LBP lasting longer than 3 months duration within the past 2 years and were not required to be in pain at the time of testing. All participants were screened for contraindications to spinal manipulation by performing an orthopedic and neurological examination. Specific exclusion criteria for all participants were a numerical pain score that exceeded 7/10 on the day of testing, radicular pain experienced below the knee during the orthopedic exam, absence of reflexes or decreased sensation/weakness below the knee during the neurological exam. Each participant provided written, informed consent before the start of each data collection session.

### Instrumentation

**Fig. 1:**
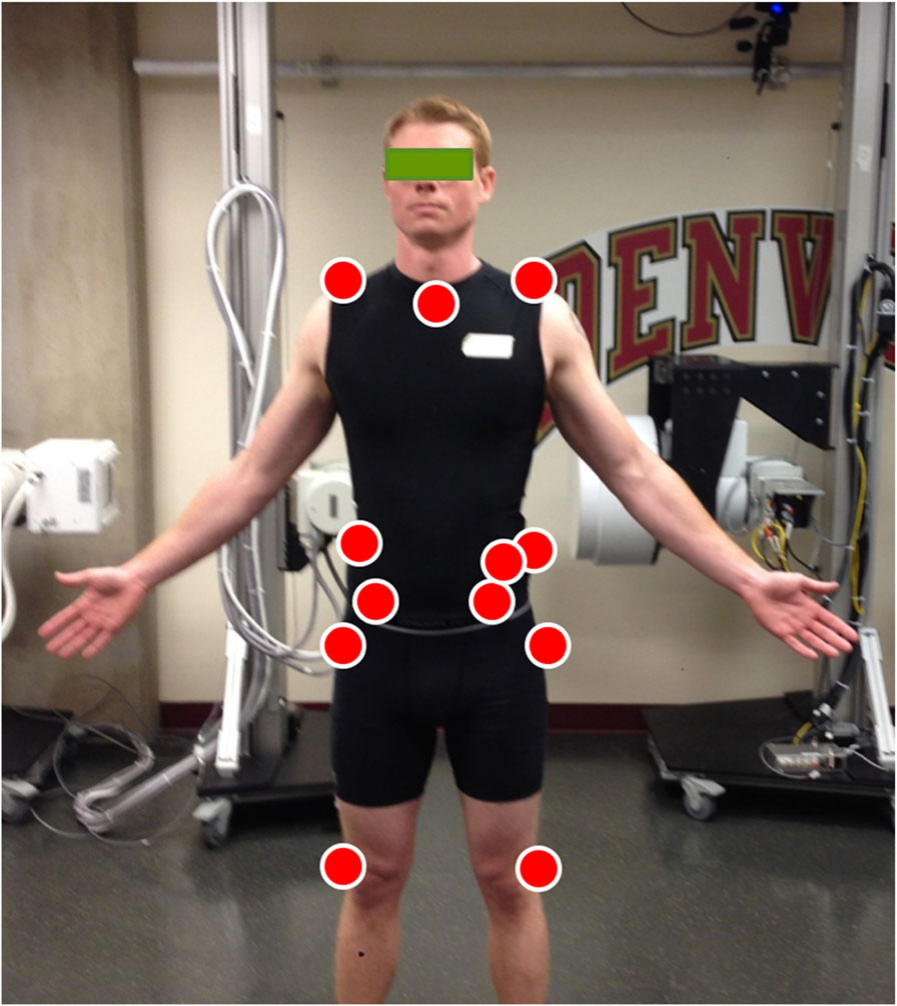
Anterior view of placement for kinematic instrumentation used to monitor pelvis and thorax movements during the sit-to-stand task

An 8-camera passive optoelectronic motion capture system (Vicon Motion Systems Ltd., Centennial, CO, USA) was used to monitor three-dimensional kinematics of the pelvis and thorax. Individual reflective markers (14 mm diameter) were adhered, using double-sided tape, to the skin overlying anatomical landmarks on the pelvis and thorax. Markers were positioned bilaterally over the acromion processes, the iliac crests, anterior superior iliac spines and the posterior superior iliac spines. Individual markers were also positioned over the spinous processes of the seventh cervical (C7) and tenth thoracic (T10) vertebrae, the suprasternal notch, and on the left ilium just anteroinferior to the iliac crest (Fig. 1). The C7 landmark was identified by palpating the vertebral prominens during active neck extension. The spinous process for T10 was determined by palpating the lowest ribs and tracing back toward the spine to locate the spinous process of the twelfth thoracic vertebra and counting 2 spinous processes superiorly. All kinematic data were sampled at 100 Hz.

### Protocol

**Fig. 2:**
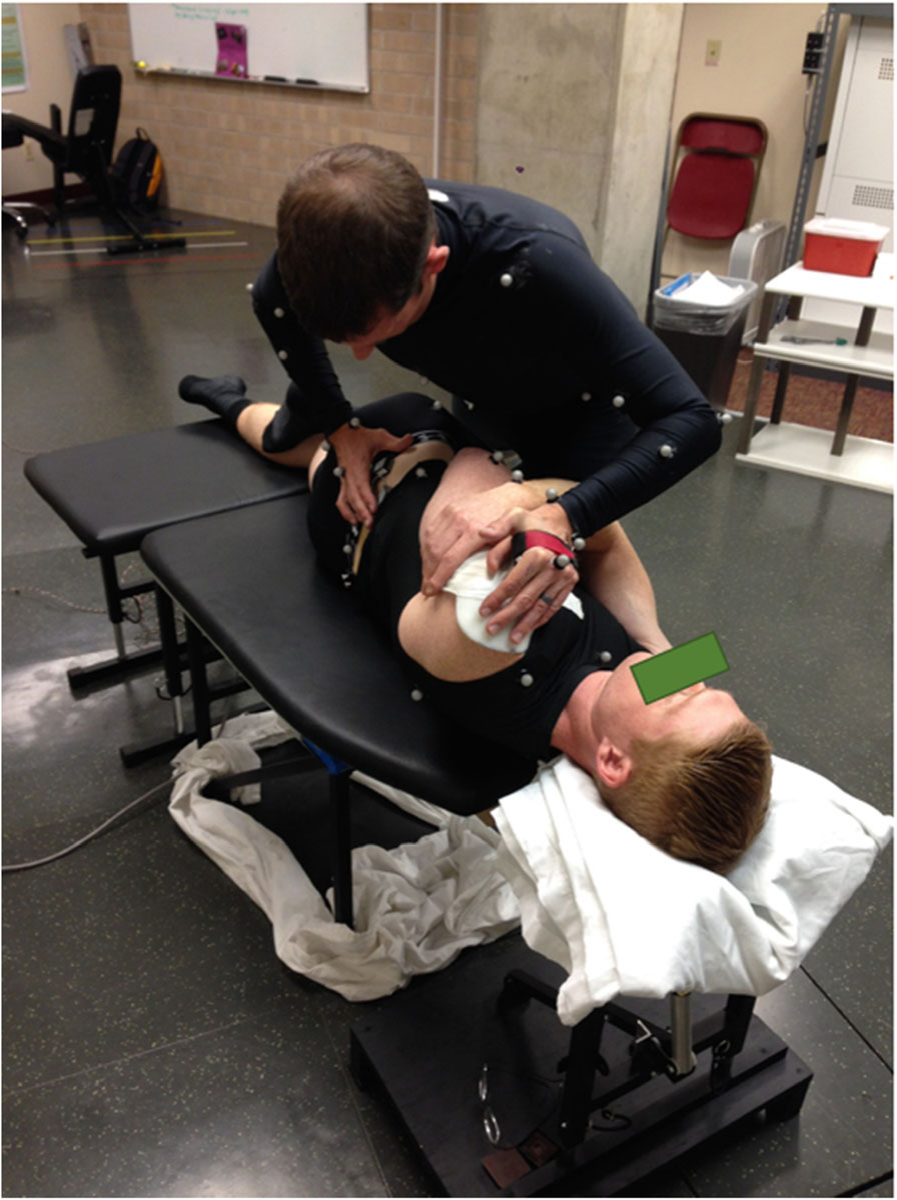
Positioning of the chiropractor and the participant during the MT interventions

Following instrumentation, participants performed a single trial of upright standing. Participants then completed a trial of the STS task before and after receiving an MT intervention. The MT intervention consisted of two spinal manipulations with a high-velocity, low amplitude impulse and two grade IV mobilisations. Spinal manipulations were characterised by a single quick force applied to the target area. Mobilisations consisted of 5 contiguous and slower cycles of a lower amplitude force applied to the target area at a frequency of approximately 1 cycle per second. All MT procedures were performed with the participant in a side-lying posture, and the clinician used a hypothenar contact to direct force to the third lumbar (L3) or first sacral (S1) vertebrae (Fig. 2). Manual therapy interventions were performed by two different chiropractors, each with more than 10 years of clinical experience. Each of the four individual treatments were separated by one to 3 min and presented to the participant in a randomized order. The L3 and S1 targets were selected because they maintained the safety of the instrumentation during the procedure.

For the STS task, participants were seated on a backless chair with their feet flat on the floor. The chair’s height was not adjusted for individual participants and their foot placement was not marked or constrained for either of the STS trials. The following instructions were provided to each participant before the STS trials: “While you are seated, please cross your arms over your chest. Now stand up.” Participants proceeded to perform the STS task at a self-selected pace and were not provided with an opportunity to practice the movement. The task was successfully completed once the participant achieved an upright standing posture.

### Data processing

Three-dimensional kinematic data from the individual markers were imported into Visual3D (C-Motion Inc., Germantown, MD, USA) for post-collection processing. Anatomical frames of reference for the pelvis and thorax were defined from the upright standing trial. Movements of the pelvis and thorax were tracked during the STS trials using markers affixed to each segment. Lumbar spine angular deviation was defined as the relative movement between the pelvis and thorax, which was determined using an Euler decomposition sequence of flexion/extension, lateral bend, and axial rotation [36]. Lumbar spine angular velocities were also derived from the kinematic data. Velocity time-series data were used to visually identify and manually select the frames for initiation and termination of the STS task.

**Fig. 3:**
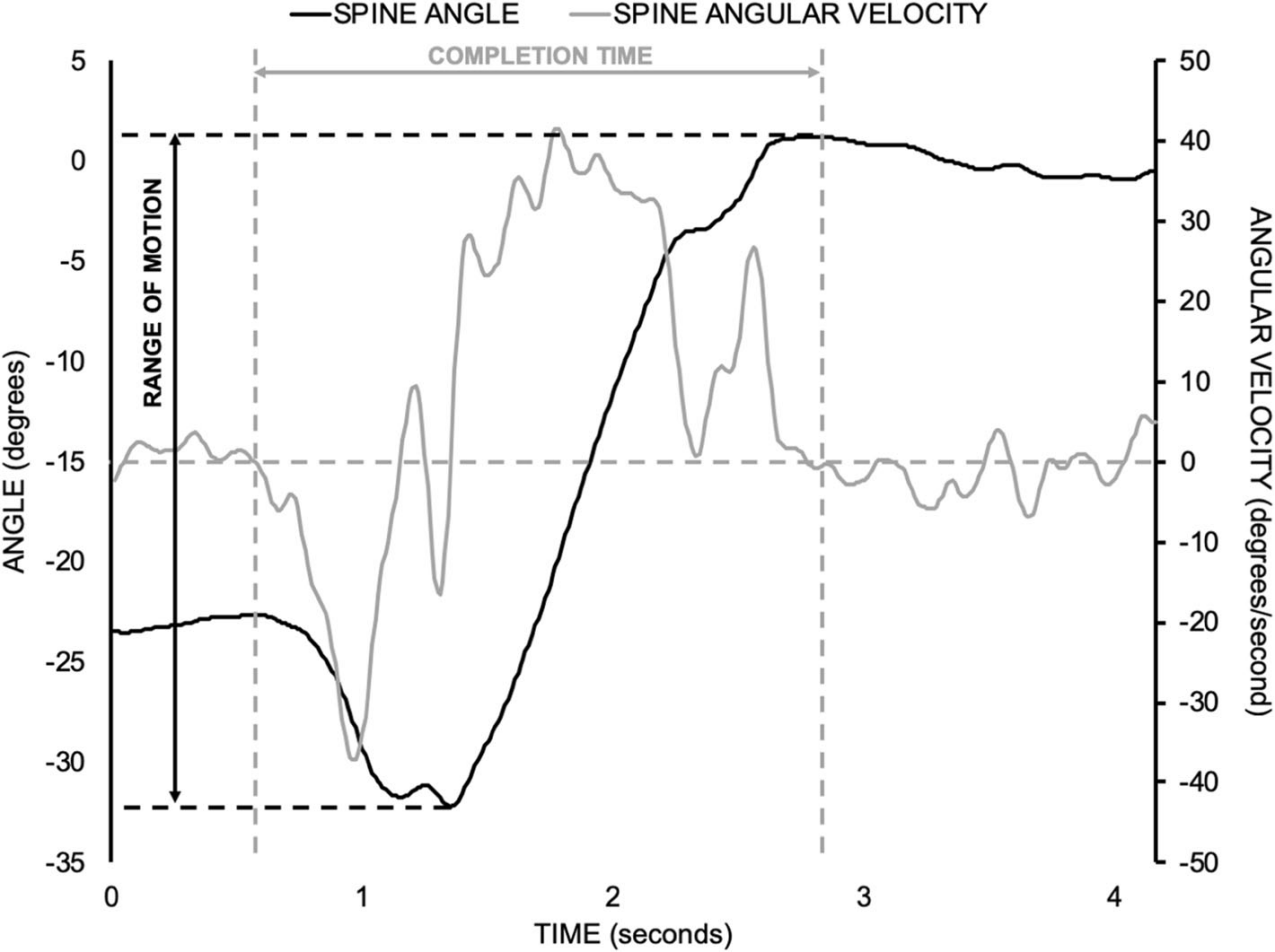
Sample time-series data of the spine angular position (black solid line) and velocity (gray solid line) in the sagittal plane during a single trial of the sit-to-stand task. Vertical gray dashed lines denote the identified instants for initiation and termination of the sit-to-stand task. Task completion time was the difference between the termination and initiation timepoints. Horizontal gray dashed lines denote the maximum and minimum sagittal plane spine angles that occurred during the sit-to-stand task. Total spine range of motion was determined as the difference between the identified maximum and minimum spine angles

Two dependent measures were derived from each STS trial to evaluate performance (Fig. 3). The first was the time to complete the STS task, which served as a functional measure of performance. Total lumbar ROM in the sagittal plane during the STS task was used as a biomechanical measure of performance [37, 38]. Given the preliminary nature of the current investigation, the proportion of participants whose pre-post changes exceeded the standard errors of measurement (SEM) was calculated for each dependent measure. Standard error of measurement for completion time of a single STS movement was 0.5 s, which was estimated from the previously reported standard error of measurement for the 5 cycle STS test (4.2 s) in participants with chronic non-specific LBP [39]. A reported standard error of measurement of 3.4 degrees for utilised lumbar sagittal plane ROM during an STS task performed by participants with chronic non-specific LBP was also used [38]. Validity of these SEM estimates was limited since they were obtained from studies with different populations and protocols. As mentioned above, these SEM estimates were used to assist with interpreting group- and individual-level changes in STS task performance after the MT intervention.

### Statistical analysis

All statistical analyses were performed using SAS (Cary, NC, USA). Group descriptive measures (e.g. means, standard deviations) were determined for the participant demographic data, as well as the functional and biomechanical dependent measures from the STS trials. The functional and biomechanical dependent measures from the STS task from participants with either acute or chronic LBP were combined to form a single LBP group for inferential statistical analysis. Kolmogorov-Smirnov tests were performed and confirmed that the distributions of dependent measures were not statistically different from a normal distribution. Levene’s tests were also performed and statistically confirmed equality of variances between the paired samples. Thus, pre-post differences for the time to complete the STS task and the utilised spine sagittal plane ROM during the STS task were statistically evaluated by paired samples t-tests. Statistically significant changes were identified for any *p*-value that was less than 0.05. Effect sizes were determined using Cohen’s d using the mean and standard deviation of the individual pre-post differences. A bootstrapping procedure, using 200 samples, was implemented to determine the 95% confidence intervals (CIs) for the effect size [40].

## Results

### Participants

Kinematic data were obtained from 40 participants; however, data from 5 participants were not included in the analysis due to missing marker data during the STS trials that prevented tracking of the pelvis and/or thorax. All participants reported a level of pain greater than 0 on the day of data collection, and only 2 participants in the group with chronic LBP reported a level of pain that was less than 2. Demographics for the sample of participants are summarised in Table 1.

**Table 1:** Demographics of participants with usable datasets. Standard deviations are presented in parentheses. NRS = Numerical Rating Scale

	SEX (M/F)	HEIGHT (cm)	MASS (kg)		NRS (/10)		AGE (years)	
ACUTE	10/8	174 (8)	77.7	(13.3)	3.1	(0.7)	29.0	(7.8)
CHRONIC	9/8	168 (11)	71.1	(15.2)	3.6	(1.5)	36.1	(10.1)
ALL	19/16	171 (10)	74.5	(14.7)	3.3	(1.2)	32.4	(9.7)

### Sit to stand

**Fig. 4:**
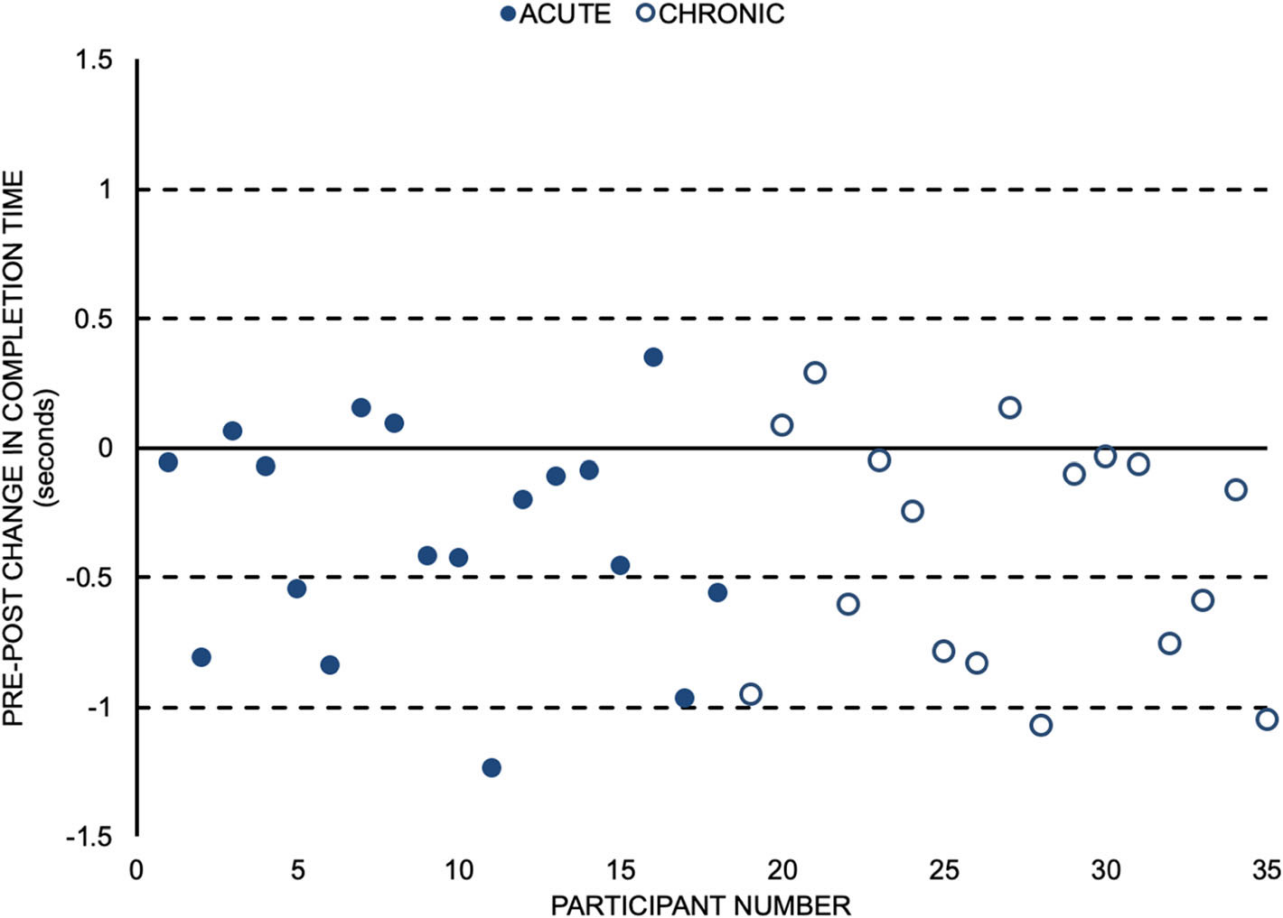
Change in time to complete the sit-to-stand task following the manual therapy intervention for individual participants. Closed circles represent acute low back pain participants, and the open circles represent chronic low back pain participants. The dashed lines represent integer multiples of approximations of the standard error of measurement from a 5 cycle sit-to-stand test: 0.5 s each

A total of 28/35 (80%) participants required less time to complete the STS task after the MT intervention (Fig. 4). From those participants who improved their completion times, 14 (40% of the sample) of them improved their task completion time by more than 0.5 s. On average, participants completed the STS task in less time (mean reduction of 0.4 s) following the MT intervention (d = 0.84, 95% CI for d = (0.57,1.18); *p* < 0.001) (Table 2).

**Table 2:** Descriptive and inferential statistics for the time to complete the STS task and the utilised lumbar sagittal range of motion (ROM) before and after the manual therapy intervention. Standard deviations for pre- and post-intervention group averages are presented in parentheses. Values in parentheses beside the effect size estimates (d) represent the upper and lower limits for the 95% confidence interval of the effect size

	PRE	POST	d	*p*
COMPLETION TIME (seconds)	2.7 (0.6)	2.3 (0.5)	0.84 (0.57,1.18)	< 0.001
LUMBAR SAGITTAL ROM (degrees)	24.5 (8.7)	27.2 (9.8)	0.48 (0.11,0.84)	0.007

**Fig. 5:**
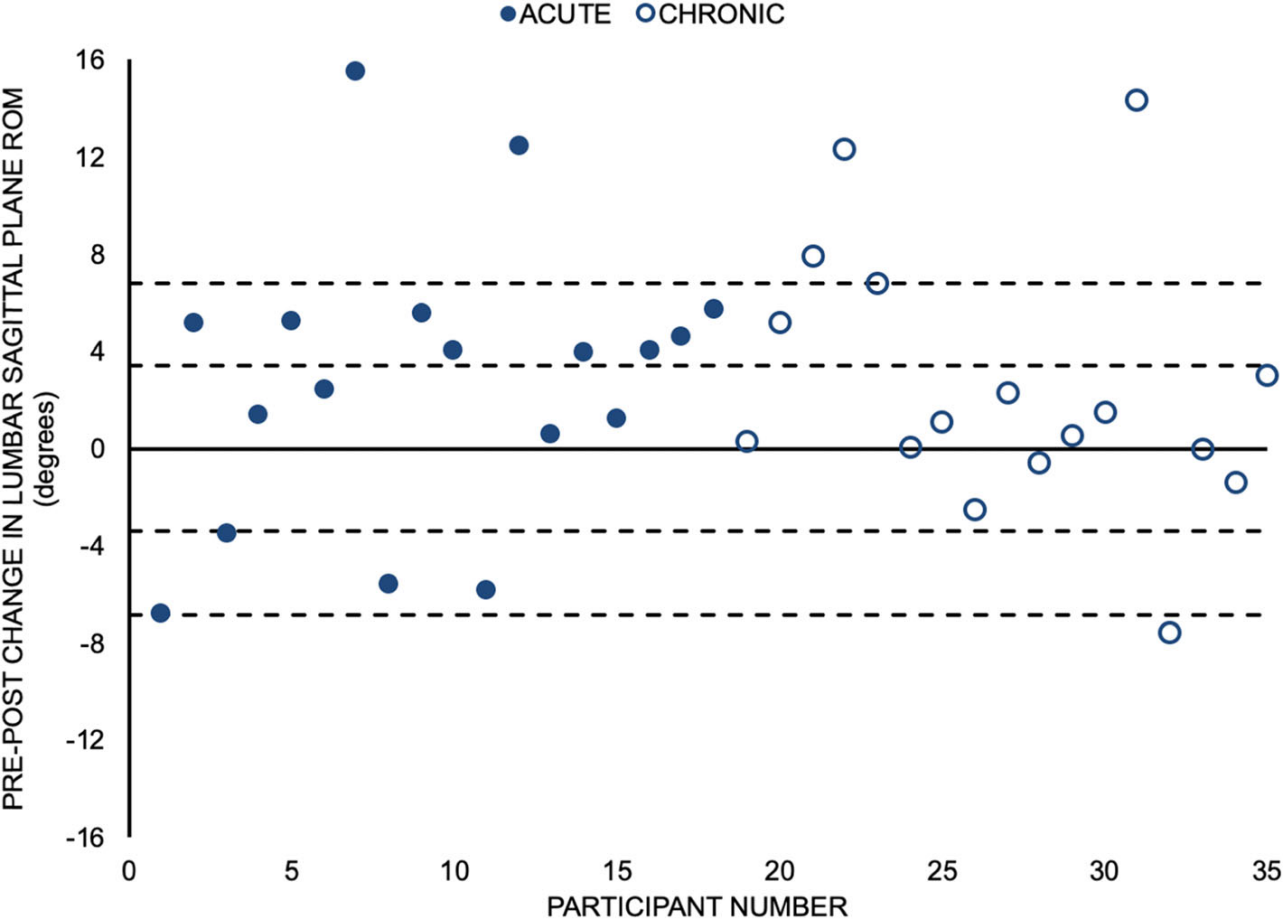
Change in utilised sagittal plane lumbar spine range of motion (ROM) during the sit-to-stand task following the manual therapy intervention for individual participants. Closed circles represent acute low back pain participants, and the open circles represent chronic low back pain participants. The dashed lines are integer multiples of approximations of the standard error of measurement that were obtained from the literature: 3.4 degrees each

Utilised sagittal plane lumbar spine ROM was greater for 26/35 (74%) participants after the MT intervention (Fig. 5). A total of 15/35 (43%) participants increased their utilised ROM by more than 3.4 degrees. Overall, a statistically significant increase in spine flexion ROM (mean increase of 2.7 degrees) utilised during the STS task was observed following the MT intervention (d = 0.48, 95% CI for d = (0.11,0.84); *p* = 0.007) (Table 2).

## Discussion

The current investigation sought to determine if biomechanical and functional performance of an STS task was altered following a MT intervention in participants with either acute or chronic LBP. Our findings demonstrated that participants with LBP utilised a greater lumbar ROM in the sagittal plane while performing the STS task and the time to complete the movement decreased after a MT intervention that combined mobilisations and spinal manipulation directed to the lumbar spine and pelvis. This is preliminary evidence that performance of functional movement tasks by patients with LBP may be acutely altered following MT intervention targeted toward the lumbar spine and pelvis.

A collection of neuromechanical investigations suggesting a possible effect of MT on spine movement can help explain the present results. However, conflicting findings have been reported between studies measuring movement outcomes that reflect a person’s active movement capacity in non-functional contexts (e.g. planar ROM) following either spinal manipulation or mobilisation [7, 18–20]. Manipulation directed toward the cervical spine can influence sensorimotor integration within the central nervous system [12]. Other work has demonstrated an acute increase in motor unit excitability and cortical drive to the soleus muscle following spinal manipulation [13, 14] as well as facilitating activation of the lumbar multifidus [16, 17]. An increased magnitude of internal oblique activity during a rapid arm raising task has also been reported following mobilisation [11]. Mechanically, a greater reduction of the spine’s passive stiffness in the posteroanterior direction has been observed amongst patients with LBP that report an improvement in disability following spinal manipulation applied to the low back and pelvis [15, 16]. The observed increase in ROM utilised by participants with LBP during the STS task following the MT intervention suggests that the aforementioned neuromechanical changes could manifest as alterations to the performance of functional activities that require a submaximal amount of spine movement. Using previous work comparing STS performance between participants with and without LBP, the increased ROM and reduced completion time each represent changes toward improved STS performance following MT [33, 39, 41, 42]. This remains speculative given the study’s limitations (described below) but provides a foundation for future work investigating the impact of MT on performance of functional tasks.

The STS task is a functional movement since it is a multi-joint and multi-planar movement performed an average 60 times per day, and it is relevant since patients with LBP commonly report difficulty rising from a chair [24, 25, 31]. Clinicians and researchers often utilise the STS task as a way of evaluating function in patients with LBP. Quantitative studies have reported differences in several biomechanical variables during performance of the STS task between participants with and without LBP. Collectively, these studies have reported that participants with LBP tend to perform the STS task with: smaller ROM in the lumbar spine [33, 38]; lower flexion and extension velocities of the lumbar spine [33]; delayed onset of pelvic movement during initiation [43]; interjoint coordination between the lumbar spine and hips that favors less hip lag at the initiation and greater hip lead at the termination of movement, as well as increased relative phase and greater variability of relative phase at the initiation and termination of movement [33, 34]; less concentric muscle power [42]; and, greater energy demand and less efficient performance [44]. Functionally, patients with LBP require more time to complete 5 consecutive repetitions of the STS task [32]. There is potential for using measurements related to the movement within the clinical environment as the introduction of low-cost devices capable of capturing time-varying movements and forces expands [45, 46]; however, a recent review determined that the current clinical utility of kinematic and kinetic measures for patients with LBP is limited to observational analysis [22].

A few limitations of the study’s design and population must be considered when interpreting the findings from this work. First, the current study used a pre-experimental single group pretest-posttest design without a control group or randomization. The second limitation is related to the combined use of manipulation and mobilisation as the intervention. Both of these limitations preclude making strong conclusions on the direct effects of spinal manipulation or mobilisation on the biomechanical or functional performance of the STS task in patients with LBP. The combination of participants with acute and chronic LBP within a single group is another limitation to be addressed by future work. Furthermore, we did not exclude participants on the basis of diagnosed lower extremity pathology (e.g. hip pathology). This is relevant considering that the STS task is used to assess lower extremity function and its outcomes could be influenced by lower extremity pathology [47]. It is important to reiterate that each participant underwent an orthopedic and neurologic examination to screen for contraindications to receiving spinal manipulation, which included radicular symptoms below the knee and/or the absence of reflexes, decreased sensation or weakness below the knee. As a result of these limitations, our purpose, hypothesis and conclusions have been restricted to comment upon changes in the biomechanical and functional performance of the STS task that were observed in participants with LBP following the MT intervention. Additionally, because this study investigated whether there was an immediate effect on the STS after MT, further work will need to investigate whether the changes persist or result in clinical changes.

There are additional limitations related to the implementation of the STS task in the current investigation. For example, the use of a single STS trial before and after the MT without any prior practice introduces the possibility for observed changes in the functional and biomechanical outcome measures to be the result of participant learning. While this is a possibility, the aforementioned commonness of the STS movement in daily life [24] would suggest a minimal learning effect. A related limitation is the possibility that observed changes in the two outcome measures after the MT intervention could be attributed to trial-to-trial variability within a participant. The decision to use a consistent chair height for all participants is another limitation, considering that chair height has been identified as a key determinant of STS task performance [48]. The within-subjects design of the current study ensures that limitations related to chair height would have been consistently represented in the STS trial performed before and after the MT intervention.

## Conclusion

In conclusion, the current investigation provides preliminary evidence to demonstrate that the biomechanical and functional performance of an STS task by populations with LBP may acutely be altered following a MT intervention. The precise mechanism remains unknown; however, it is possible that changes in performance of a functional movement such as STS might be related to a combination of altered muscle activation strategies and vertebral joint stiffness previously reported. Our findings can support the development of future hypothesis-driven work directed toward investigating the potential impact of MT on performance of functional tasks in populations with LBP.

## Data Availability

The datasets used and/or analysed during the current study are available from the corresponding author on reasonable request.

## Abbreviations

LBP: Low back pain
MT: Manual therapy
ROM: Range of motion
STS: Sit-to-stand
